# The outcomes of a mobile just-in-time-learning intervention for teaching bioethics in Pakistan

**DOI:** 10.1186/s12909-022-03698-9

**Published:** 2022-09-13

**Authors:** Azra Naseem, Sameer Nizamuddin, Kulsoom Ghias

**Affiliations:** 1 Institute for Educational Development, Aga Khan University, IED-PDC, 1-5/B-VII, F. B. Area, P.O. Box 13668, Karachi, 75950 Karimabad Pakistan; 2 Medical College Dean’s Office, Aga Khan University, Stadium Road, P.O. Box 3500, Karachi, 74800 Pakistan; 3 Werklund School of Education, University of Calgary, 2500 University Drive NW, Calgary, AB T2N 1N4 Canada; 4 Department of Biological and Biomedical Sciences, Aga Khan University, Stadium Road, P.O. Box 3500, Karachi, 74800 Pakistan

**Keywords:** Bioethics, Just-in-time-learning, Mobile application, m-learning

## Abstract

**Introduction:**

The study aimed to test the effectiveness and the feasibility of a mobile just-in-time-learning (m-JiTL) approach for teaching bioethics at a university in Pakistan. Over four months, a mobile app (EthAKUL) was used to enhance ethical reasoning among practising nurses, trainee physicians, and medical and nursing students utilising the m-JiTL approach. Participants used EthAKUL to access bioethics modules and participate in asynchronous discussions.

**Methods:**

A mixed methods design was adopted. Pre- and post-knowledge tests were used to assess changes in participants' knowledge of bioethics concepts, while pre- and post-surveys were used to assess changes in participants' attitudes towards m-learning. After the intervention, focus group discussions with the participants were held. Analysis of the discussion posts and meeting notes was conducted.

**Results:**

The learners had a favourable attitude toward using mobile devices for learning purposes at the start of the intervention, and the score remained positive afterwards. Bioethics knowledge test scores improved at the end of the intervention, with medical students experiencing the greatest improvement. However, because of the high drop-out rate and lack of participation after the initial phase, it is unclear whether the increase in score or positive attitude is the result of the intervention, making it difficult to draw firm conclusions about the intervention's success.

**Conclusions:**

EthAKUL is the first of its kind app for teaching bioethics, and the study has offered important insights into adopting new pedagogies and technologies for bioethics teaching. It has also identified issues with the design of the app and m-JiTL pedagogy that must be addressed before curriculum-wide adoption.

## Introduction

The primary goal of teaching bioethics is to improve the ability of healthcare practitioners to care for patients and their families. Clinical situations are frequently complex and critical, and healthcare professionals often must make difficult medical decisions involving patients and their family members. In all situations, it is imperative to be respectful of patients and their wishes and make clinically sound and ethical decisions [1]. Bioethics is essential in the advocacy of the relationship between scientific practice and values essential to people’s life and society, and teaching bioethics enables students to understand the conflicting areas and develop decision-making skills based on moral and ethical principles [2, 3]. There is no resource for identifying the best option in real-time ethical decision-making. The dynamic interaction of theory and practice, experience and reflection can help healthcare professionals work constructively to manage ethical challenges in clinical settings while maintaining everyone’s dignity and respect [1]. Therefore, ethics education should be about a process that embeds ethics in learners’ minds and practice [4] and enables ethical decision-making.

Developing countries in Asia and Africa have been late adopters of bioethics education in general. Many medical colleges in less well-resourced countries still lack formal bioethics curricula or the means to implement them [5–8], while it is in these contexts perhaps that bioethics training is most needed [9–11]. In Pakistan, for example, weak socio-political systems and infrastructure have exacerbated health inequities. The public health system is largely unregulated, poorly funded, and ill-equipped to cater to a vast population that lives below the poverty line. Quality medical care is available in some private sector institutions, but access is limited for most of the population. Ethical dilemmas in healthcare practice are commonplace and in the absence of strong regulatory mechanisms and legal frameworks, issues of governance and integrity arise often and go largely unresolved. Bioethics education that develops and hones ethical decision-making skills is critical for healthcare practitioners in such settings [12]. Moreover, ethics education in medical school lacks interprofessional training and participation. With the contemporary challenges arising in healthcare systems, social movements, and technological advancements, it is even more critical and urgent to find effective ways to implement bioethics curricula in health professions education [2].

Mobile devices, such as smartphones and tablets, along with the just-in-time teaching and learning (m-JiTL) approach can support healthcare professions education [13–15]. Previous research has reported positive outcomes of mobile learning (m-Learning) [13, 16] and predicted a rise in the use of mobile devices in medical education and practice [17, 18]. Medical school and healthcare leaders are encouraged to set the agenda to maximise the benefits of m-Learning while avoiding unexpected consequences, such as superficial learning [18]. Efficiency in acquiring new information [19], portability, resonance with life context and the stage of residency were found to influence residents’ usage of m-Learning, as were the trustworthiness of information received and the relevancy of the material [20]. m-Learning has been used in undergraduate and graduate medical education, such as ECG technique knowledge and test performance [21], teaching and learning of genital system anatomy [22], as well as accessing research journals and medical dictionaries, and using disease and drug management apps [23]. The past perception studies on m-learning have shown it to be useful in clinical settings as it encourages flexible communication with faculty and peers regardless of time and space and provides easy access to resources for learning and clinical care [18, 23–25].

Just-in-time learning (JiTL) is an approach that relies on technology for providing with greater learner control; time and place independence; and the use of Internet based information [26], and JiTL solutions can be used to meet the demand for on-the-job learning [27]. Combined with mobile technology, the m-JiTL environment takes account of the mobility of technology, learners, and learning [28], and is particularly suitable for interprofessional education where medical and nursing students and healthcare workers can study together. It affords quick timely communication and collaboration amongst learners and teachers. Prompt feedback plays a critical role in the learning process and with the m-JiTL approach queries can be answered just-in-time, making it easier for students and trainees to put their learning into action to benefit patients. Mobile devices can also be efficient tools to conduct assessments, rather than spending hours in classroom exams [15, 29].

While studies have reported various innovative methods of teaching bioethics, for example, using art [30] and virtual learning object [31], there is limited evidence in support of using m-JiTL in clinical bioethics teaching. However, research from the field of medical education broadly suggests that this approach could be beneficial for bioethics instruction by enhancing learners’ comprehension of bioethics principles and their application in context, that is during their clinical experience, instead of didactic lectures [32].

Bioethics teaching is a part of the medical and nursing education at the private university in Pakistan where the study reported in this paper was implemented. Despite being an early adopter of bioethics instruction, bridging the gap between theory and practice has been a challenge for the faculty, and the need for exploration of innovative methods is recommended [33]. In this context, the study presented here is the first of its kind incorporating m-JiTL for bioethics teaching with interprofessional facilitators and learners. This study plays a significant role in filling the scholarly gap and guiding future research and policy initiatives in mobile learning in medical education. The purpose of the study was to evaluate the effectiveness of a mobile application called EthAKUL designed [34] and used in the intervention for JiTL of bioethics to medical and nursing students and trainees at a private university in Pakistan. Since this was a new teaching approach being tested at the university, a small-scale preliminary intervention was planned to study the participants’ experiences of using the mobile app, including challenges and successes, and changes in participants’ understanding of bioethics topics and attitude towards m-learning were assessed.

## Methods

The overall aim of the study was to evaluate the effectiveness of EthAKUL and determine the feasibility for curriculum-wide adoption. Specifically, the study focused on determining changes in learners’ knowledge of bioethics and attitudes towards m-Learning at the end of the intervention, any differences based on gender and discipline, and exploring participants’ experiences of using EthAKUL focusing on the most frequently used features, benefits, and challenges of using m-JiTL approach for teaching bioethics.

The research questions were:

1. Do learners’ scores change from the pre-test to the post-test? Do gender and discipline differences influence learners’ test scores?
2. What changes can be seen in learners’ attitudes toward mobile learning as measured through the pre and post intervention attitude scale?
3. What were the usage patterns and which EthAKUL features are most frequently used, and why?
4. What are the participants’ experiences of teaching and learning using EthAKUL, including successes and challenges?
5. What do the participants perceive as the future of EthAKUL?

### Design

The quantitative data were collected through pre-and post-questionnaire and knowledge tests, whereas the qualitative data were collected through focus group discussions, discussion logs, and responses to open-ended questions about experiences. Mixed methods triangulation approach was used to combine the quantitative and qualitative data for an in-depth analysis of the outcomes of a m-JiTL app for bioethics [35–37]. Integration of methods occurred at the design (i.e., intervention mixed methods), method (i.e., embedding) and at the interpretation and reporting (i.e., narrative) levels.

All three authors were participant-researchers in the intervention with KG also serving as a facilitator. Each researcher brought their unique experiences and ideas due to their varied disciplinary backgrounds in medical education (KG), ethics education (KG, SN), faculty development (AN, KG) and e-learning (AN, KG). One of the researchers (SN) is also a graduate student. As participant-researchers, we were aware of our expertise, biases and assumptions and the potential bias that could result from the interaction between faculty-as-researchers and students-as-participants, and our peers as participants. Reflexivity allowed us the space to explore how our individual roles and assumptions were affecting the research process and our interpretations. As a result, the qualitative interviews and focus group discussions were conducted by a researcher who was hired specifically for this purpose and had no teaching responsibilities (SN). This allowed learners to freely express their opinions about the difficulties they encountered during the intervention. Similarly, the qualitative data (including discussion posts) were analysed by two researchers (AN, SN) who were not intervention facilitators. We also discussed our interpretations with people who were both familiar and unfamiliar with the research context in order to ensure the validity of our findings.

### Setting

The study was conducted at a private university in Pakistan, which offers medical and nursing education programmes that incorporate bioethics teaching. Specifically, bioethics teaching spans across all five years of the undergraduate medical education programme that has 100 students in each year and utilises multiple pedagogies for effective student engagement [12]. The curriculum includes history of bioethics, ethical principles and theories, rights of patients and vulnerable groups, ethical issues in reproductive health, palliative care, research ethics, among other relevant topics. Bioethics teaching is complemented by longitudinal themes such as communication skills and professionalism, which are essential for ethical practice. The four-year Bachelor of Nursing programme, with about 150 students in each year, includes a 2.0 credit hour course titled Nursing Ethics which aims to provide awareness on general ethical principles and theories. These are then built upon in the clinical courses as an integrated learning outcome. There are over 500 postgraduate medical education trainees in 34 accredited residency programs. All residents are required to attend two mandatory workshops on clinical ethics as part of their core curriculum, one in Year 2 of training and the second in Year 4. The workshops use a case-based approach to identifying ethical dilemmas. The medium of instruction across all educational programmes in the institution is English.

### Participants

Due to the server’s capacity, the app could enrol a maximum of 100 users (including students, facilitators, and the researchers). Undergraduate and postgraduate medical and nursing students, residents, and practising nurses were invited to join the study through emails and posters on the bulletin boards in the medical college and school of nursing between September – October 2018. The inclusion criteria included participants who: 1) owned a smartphone, 2) could converse in English, 3) were willing to join the study voluntarily, and 4) belonged to the university. The study enrolled 67 learners from diverse specialities who matched the inclusion criteria. A total of 26 faculty members who were actively involved in ethics-related activities at the institution, including teaching and consults, were invited. Sixteen, including the research team, volunteered to join as facilitators. They were from various specialties, such as internal medicine (*n* = 3), basic sciences (*n* = 1), psychiatry (*n* = 1), surgery (*n* = 3), emergency medicine (*n* = 1), community health sciences (*n* = 2), paediatrics (*n* = 1), oncology (*n* = 1), nursing (*n* = 2), and obstetrics and gynaecology (*n* = 1). Some facilitators had received formal ethics training, e.g., Masters in bioethics. Others have taken short courses on bioethics as a part of their training. All facilitators were considered bioethics experts at the university, and some were even considered regional experts in bioethics and had been involved in training for the last many years. Throughout this paper, students have been referred to as learners, and faculty members have been referred to as facilitators.

### Intervention

**Fig. 1:**
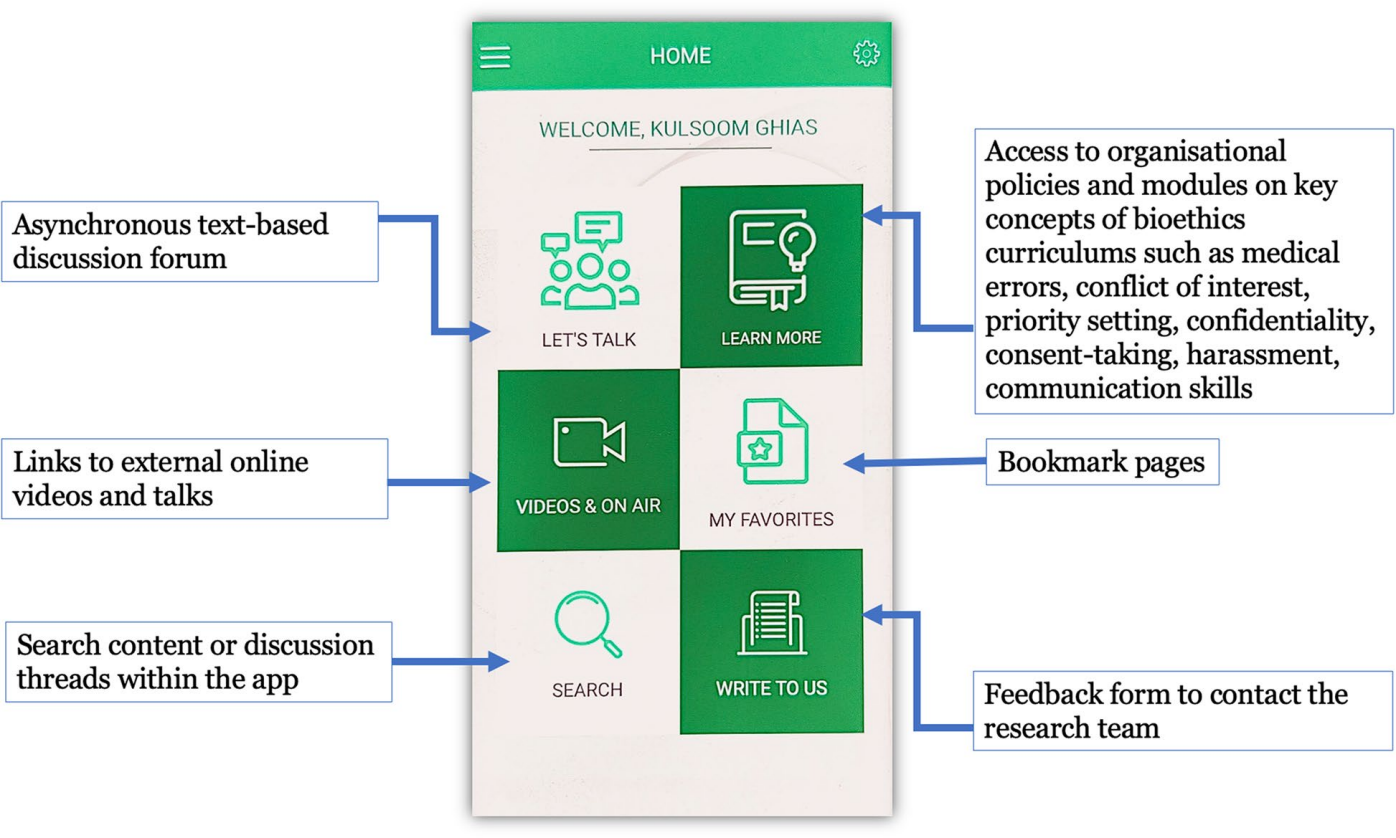
Features of EthAKUL

The study was conducted during November 2018 – February 2019. The intervention involved using EthAKUL with medical and nursing students and trainees to engage them in exploring real-life ethical dilemmas as a part of m-JiTL approach. EthAKUL is password-protected app, available for iOS and Android operating systems with the following features (Fig. 1) and the details of the app design phase are reported elsewhere [34].

The m-JiTL approach allowed learners to access the content and participate in discussions in their own time, which was important for healthcare professionals with demanding schedules.

Before the intervention, four workshops of 1.5 h each were organised to introduce EthAKUL and to collect pre-intervention data. Depending on their availability, learners attended one of four workshops in which they were introduced to the project’s aim and duration. Informed consent was taken; consenting learners downloaded EthAKUL and completed the pre-intervention data collection tools.

One orientation workshop was organised for the facilitators, where they were introduced to the research study, their role as facilitators, and the theory and practice of m-JiTL. Informed consent was obtained. An on-call schedule was devised to facilitate discussions. Each facilitator was randomly partnered with another, resulting in 8 pairs of facilitators responsible for facilitating asynchronous discussions.

At the start of the intervention, an introduction email was sent to all learners, encouraging them to submit their ethical dilemmas on the “Let’s Talk” forum. At the end of each week, an email was sent to all learners outlining the issues that had been discussed and a reminder to post further ethical dilemmas. A facilitators’ WhatsApp group was formed to assist administrative and IT-related concerns. The discussions took place in English, which is the medium of instruction at the university. During November 2018- February 2019, the learners posted ethical issues/dilemmas on the “Let’s Talk” forum and the faculty members facilitated the discussion.

### Data collection

**Fig. 2:**
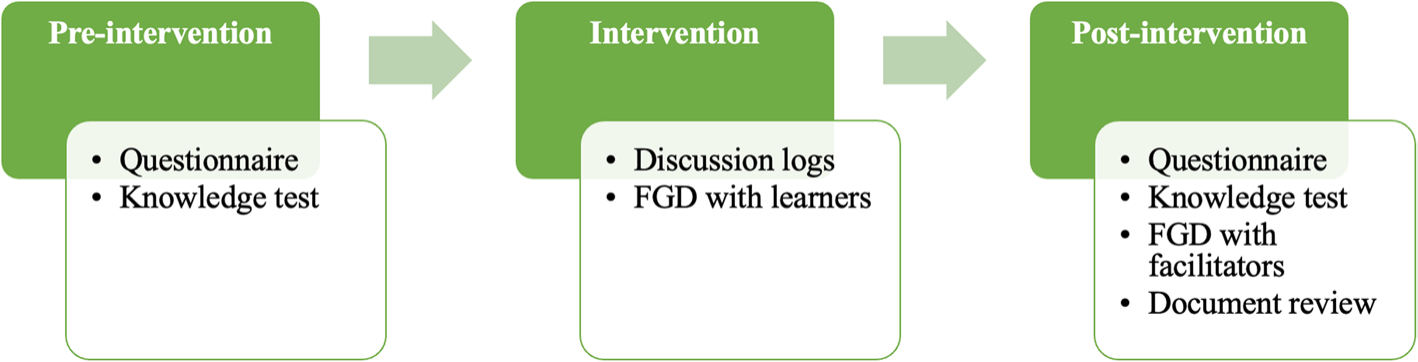
Sequence of methods

The data were collected during three stages, i.e., pre-intervention, during-intervention, and post-intervention (Fig. 2).

#### Questionnaire

For the pre-and post-intervention, a questionnaire was administered using Google forms to collect data on learners’ experiences with and attitudes about m-learning. The questionnaire had two sections. Section A featured eight questions concerning socio-demographics (name, gender, English language fluency), and learners’ year and programme of study. Section B included eight questions on past experiences with utilising mobile devices for study purposes, smartphone ownership, and perceptions about the reliability of information obtained via mobile devices adapted from [25]. In the post-intervention questionnaire, four questions were included to assess learners’ experiences during the intervention. This section also included 19 items on a five-point Likert scale (1-strongly disagree to 5-strongly agree) to assess learners’ attitude towards m-learning [24], with a score of 3 or above representing a positive attitude. The two instruments [24, 25] were adapted because of their relevance to the present study’s context. Cronbach alpha scores for pre-intervention (0.935) and post-intervention (0.909) for the attitude scale (19 items) were calculated after the scale were modified and scores suggest that the scale’s internal consistency was high [38]. The learners completed the pre-intervention questionnaire during the orientation workshops. The link to the post-intervention survey was emailed to all learners one week after the intervention ended, so they may complete it at their leisure.

#### Knowledge tests

A knowledge test was designed and administered before and after the intervention to assess changes in learners’ knowledge of bioethics. EthAKUL contained modules on seven topics: medical error, conflict of interest, confidentiality, consent taking, resource allocation, harassment, and effective communication skills. The test included 21 items with three questions on each topic. The learners completed the pre-test during the orientation workshops, whereas the post-test link was emailed one week after the intervention had ended.

#### Discussion logs

After the end of the intervention, the research team met and discussed the logs of the discussions in the “Let’s Talk” forum. Discussion posts were saved on the server from where they were copied and pasted on a word file for analysis purposes. Both the server and the computer on which data files were stored were password protected and only the research team had access to these files.

#### Focus Group Discussions (FGDs)

In the tenth week of the intervention, 15 learners participated in an hour-long FGD geared towards understanding the learners’ experiences of using EthAKUL. At the end of the intervention, two ~ 50-min FGDs were organised with the facilitators to obtain their views on the intervention. The FGDs were attended by 10 facilitators and were audio-recorded with permission.

#### Document review

Documents included notes of the five research team meetings held during the intervention. The notes were analysed to explore the issues during the intervention, and facilitators’ suggestions of how EthAKUL can be used in the future.

### Data analysis and statistical methods

All the numerical data were analysed using SPSS version 19. The descriptive analysis was done by calculating frequencies and means, and a paired sample T-test was used to compare the pre and post intervention data and determine the difference between pre- and post-bioethics knowledge test scores and pre-and post- m-Learning attitudes. The findings of the paired t-test should be interpreted with caution due to the small sample size. The qualitative data were analysed using thematic analysis. Two team members independently read the data multiple times to find recurring meanings to develop a coding scheme which were then grouped into themes. This was followed by a discussion to agree on common themes. Following the analysis of qualitative and quantitative data separately, emerging patterns were identified and converged into themes, comparing qualitative analysis with descriptive statistics, to determine usage patterns, successes and challenges encountered by participants, and future use of EthAKUL. Data were anonymised and learners’ qualitative responses are represented as roles followed by gender (e.g., Practising_nurse, female). The facilitators’ qualitative responses are represented as “F” followed by number and gender (e.g., F0, male).

### Ethical considerations

Ethical approval was granted by the Aga Khan University’s Ethics Review Committee. All participants were given written information about the study, and they gave written informed consent before joining the study. All methods were performed in accordance with the relevant guidelines and regulations. Data were anonymized and stored in a secure location.

## Results

Of the 67 learners who joined the intervention, only 29 completed the post-intervention questionnaire and the knowledge test, resulting in an overall ~ 41% completion rate. Of the 29 learners, more than half were women (58.6%), and almost half were enrolled in the BSc Nursing programme (48.3%), and the average age was 25 years. Details are provided in Table 1.

**Table 1:** Demographics

	**Pre-intervention**	**Post-intervention**
Total	67	29
Gender		
Male	19 (28.3%)	12 (41.3%)
Female	48 (71.6%)	17 (58.6%)
Programme of Study		
Bachelor of Nursing	33 (49.2%)	11 (48.3%)
Residents	10 (14.9%)	7 (24%)
MBBS	9 (13.4%)	6 (17.2%)
Practising Nurses	12 (16.4%)	4 (7%)
Others (Allied health, MSc Epidemiology)	3 (4.47%)	1 (3.44%)

The Results section is organised according to the study questions.

### Changes in learners’ knowledge of bioethics

Changes in bioethics knowledge were measured by comparing the pre and post test results. A change was noticed (*p* = 0.012) in the overall mean score of learners’ pre-intervention bioethics knowledge test (9.34 ± 2.37) and the post-intervention mean score (10.38 ± 1.98), indicating an increase in learners’ knowledge scores (Table 2). Further analysis was carried out to measure the differences related to gender or discipline. No significant change was found in the mean scores by gender. The analysis of the overall mean score for each programme showed the greatest increase was for medical students (*p* < 0.051).

**Table 2:** Changes in learners’ knowledge of bioethics

Variable	Pre (mean) ± SD	Post (mean) ± SD	*p* value
Overall (*n* = 29)	9.34 ± 2.37	10.83 ± 1.98	0.012
Gender			
Male (*n* = 12)	9.17 ± 2.37	10.83 ± 1.89	0.07
Female (*n* = 17)	9.47 ± 2.42	10.82 ± 2.09	0.092
Programme			
Residents (*n* = 7)	9.00 ± 2.82	11.14 ± 1.34	0.095
Nursing students (*n* = 11)	9.00 ± 1.61	9.91 ± 2.11	0.271
Medical students (*n* = 6)	8.83 ± 3.43	12.17 ± 1.33	0.051
Practising nurses (*n* = 4)	11.50 ± 0.57	11.25 ± 2.63	0.859
Allied Health (*n* = 1)	10.00 ± -	9.00 ± -	-

### Changes in learners’ attitude towards m-learning

The analysis of learners’ attitudes (19 items) towards m-Learning was done by analysing both pre- and post-intervention data separately, and then comparing the scores to see the differences. The overall mean score of 29 learners’ pre-intervention attitude (3.32 ± 042) and post-intervention attitude remained positive (3.17 ± 0.44), as a score of 3 or above represents a positive attitude on the 5-point Likert scale. The paired sample T-test performed to determine changes in learners’ attitudes showed that there was no significant difference (*p* = 0.231) in the pre- and post-intervention attitudes. The item-wise analysis (Table 3) showed that in the pre-intervention data, the mean score of the following two items was less than 3:

- Mobile learning would be better than classroom-based learning.
- I learn better through mobile learning strategies than through lectures.

**Table 3:** Analysis of learners’ pre-intervention and post-intervention attitude

**Items**		**Pre-intervention**		**Post-intervention**	
	N	Mean	SD	Mean	SD
Mobile learning through apps on smart devices is beneficial in medical education	29	3.59	0.568	3.52	0.509
Mobile learning tools provide me with rich resources for learning	29	3.55	0.506	3.34	0.614
Learning through smart devices is easy to monitor	29	3.45	0.632	3.52	0.574
Use of smart devices is relevant for my study program	29	3.45	0.632	3.52	0.574
Smart devices provide efficiency in learning	29	3.48	0.634	3.28	0.591
Mobile learning should be supplementary to traditional learning	29	3.48	0.509	3.28	0.797
Mobile learning needs well prepared learning materials	29	3.38	0.561	3.34	0.67
Smart devices can create a personally meaningful learning experience for me	29	3.38	0.561	3.45	0.572
Mobile learning can minimize the cost of learning for me	29	3.55	0.506	3.28	0.702
The use of mobile learning requires non-lecture-based teaching strategies	29	3.28	0.649	2.97	0.778
I would feel comfortable taking mobile learning courses	29	3.38	0.622	3.24	0.739
Using smart devices for learning will save my time	29	3.45	0.632	3.21	0.559
Mobile learning would be better than classroom-based learning	29	2.62	0.82	2.76	0.988
Mobile learning would enable me to interact with my professors more easily than in traditional learning	29	3	0.655	2.59	0.867
I learn better through mobile learning strategies than through lectures	29	2.83	0.759	2.79	0.774
Mobile learning enables me to understand the subject more than the traditional style of learning	29	3.03	0.731	2.76	0.786
I would like to have teaching–learning using smart devices	29	3.38	0.561	3	0.756
Faculty would need sufficient training to teach through smart devices	29	3.55	0.572	3.41	0.733
Students need sufficient changes in their learning styles for mobile learning	29	3.38	0.561	3.17	0.805
Overall scores		3.32	0.42	3.17	0.44

In the post-intervention data, the mean score of the above two items remained less than 3, and the following three other items also had a mean score of less than 3:

- The use of mobile learning requires non-lecture-based teaching strategies.
- Mobile learning would enable me to interact with my professors more easily than in traditional learning.
- Mobile learning enables me to understand the subject more than the traditional style of learning.

### Usage patterns and features of EthAKUL

**Fig. 3:**
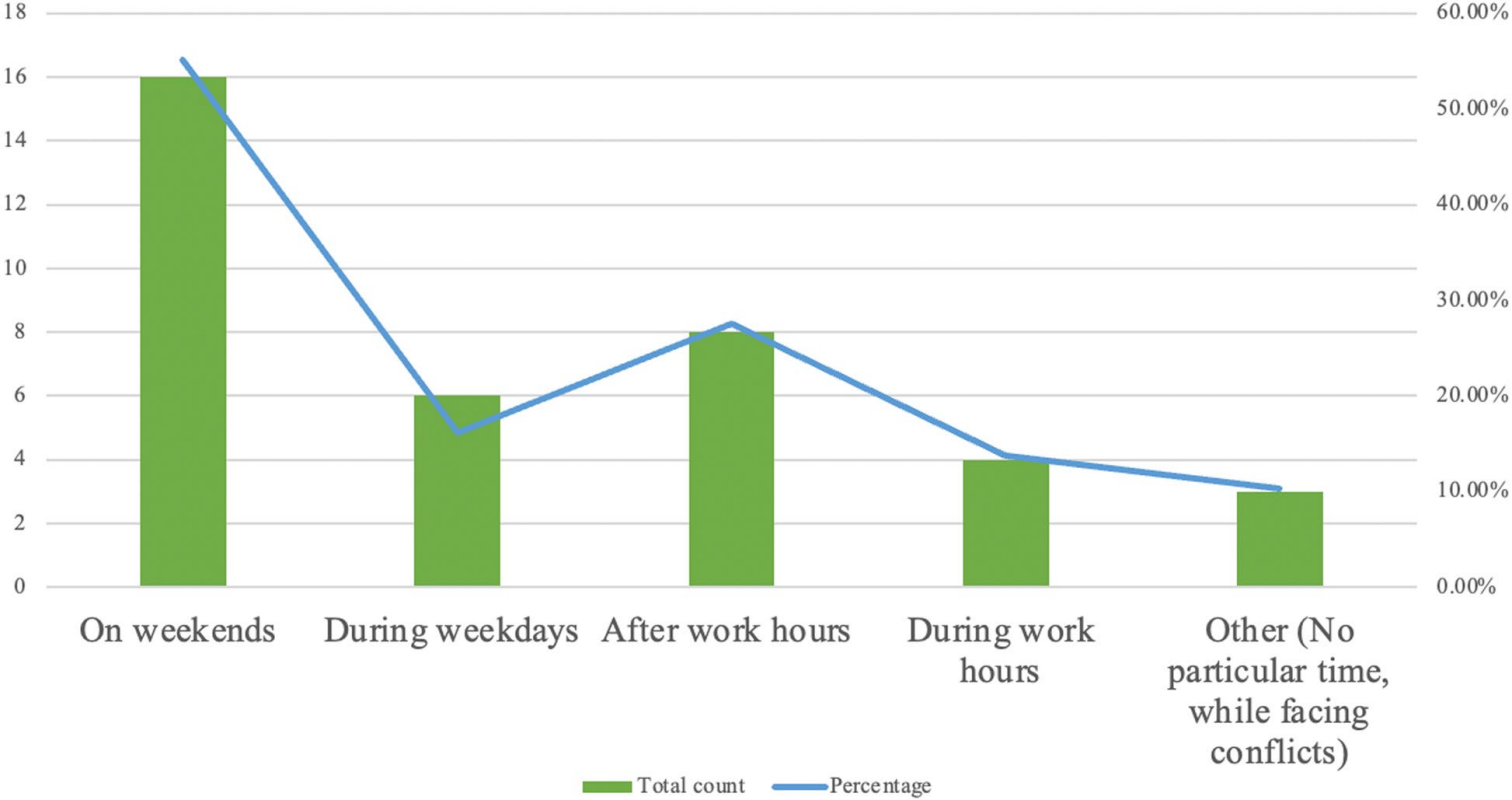
Work-day versus weekend usage

**Fig. 4:**
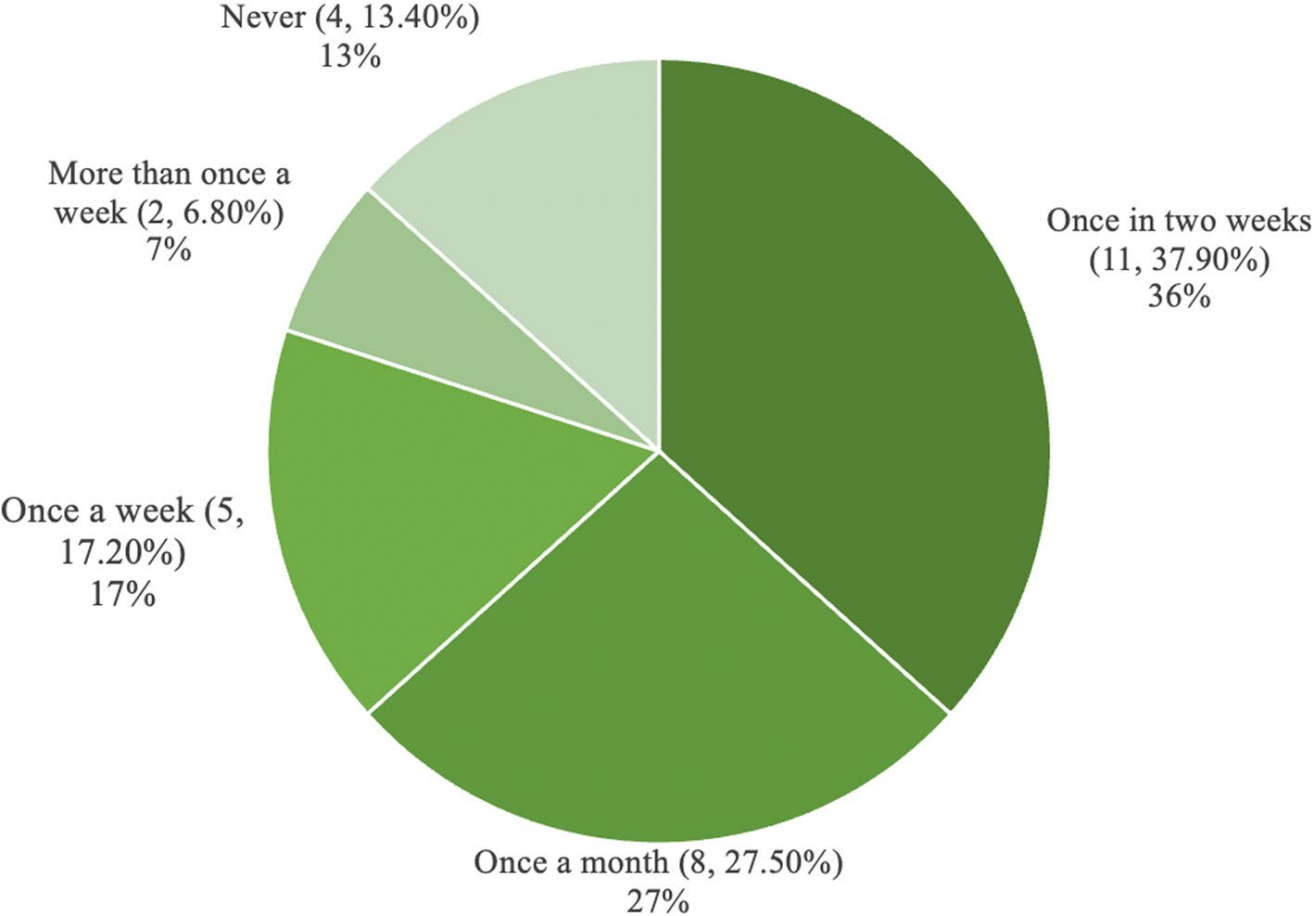
Weekly versus monthly usage

To analyse the usage patterns, we explored the questionnaire data, the number of posts on the forum and the FGDs. A large majority of the learners accessed EthAKUL on an Android phone (*n* = 22, 75.9%). Most learners (55.14%, *n* = 16) used EthAKUL on weekends (Figs. 3 and 4).

The facilitators considered EthAKUL user-friendly, even for those who were not technology savvy. They agreed that it was well-designed, allowing them to engage with learners and facilitators outside of the classroom: “It provided a much-needed virtual space, and students knew that there was somebody out there who would respond. They don’t have to wait for the teacher to meet in the classroom and discuss” (F1, Female).

EthAKUL’s most used feature was the “Let’s Talk” (58.6%, *n* = 17) discussion forum, where 25 original messages were posted, with 113 responses by the facilitators and 69 by the learners. It was followed by “Learn More”, which included organisational policies (55.1%, *n* = 16) and modules and quizzes (27.5%, *n* = 8). The learners reported that access to organisational policies related to bioethics and teaching content via “Learn More,” helped their learning. The least used feature was “Write to us” (6.89%, *n* = 2), which was an online form to contact the research team. Despite its ease of use, the following hindered the flow of discussion in “Let’s Talk” forum:

- Posts did not appear chronologically, making it challenging to find the most recent posts.
- In the linear forum all the replies related to a specific comment could not be seen together.
- No option to search for or organise specific discussion topics.
- Absence of alert notifications for new posts, making it challenging to respond on time.
- No option for audio-responses limiting those whose first language was not English.

Both facilitators and learners appreciated the anonymity feature which “allowed the freedom and comfort of posting without the fear of being singled out” (F4, Female). A learner commented “the app is a safe platform due to its anonymity” (Practising_nurse, Female). They also mentioned that the weak Wi-Fi signals inside the clinics hindered the use in the clinical areas.

Several suggestions were made to improve the app features, such as:

- “Let’s Talk” forum should be searchable, threaded and it should allow the discussion posts to be sorted according to the programme or topics.
- There should be a private space where learners can write personal reflections.
- Include a voice-note option, similar to what is present in other popular social messaging applications, to enable healthcare personnel to express ethical problems in their native language as a voice-message. This feature was important if EthAKUL were to be used “… locally or nationally, [where] there will be a lot more people who will have difficulty with the language” (F8, Male).

### Participants’ experiences of teaching and learning using EthAKUL

The analysis of the qualitative data resulted in the identification of the following themes related to the participants’ experiences of using EthAKUL.

#### Nature of posts

There were 25 initial posts in total (some examples provided in Table 4). While some were about a specific dilemma many touched upon multiple ethical dilemmas. There were no posts related to medical errors. Most of the posts discussed nurses, students and residents’ experiences working in the teaching hospital. Ethical issues with a strong cultural component to their manifestation were posted. In Pakistan, for example, younger people frequently do not challenge the older; as a result, learners commented in some posts that even when they saw bioethical principles being violated, they were not sure if they could question the faculty or their seniors. Similarly, learners emphasised the ethical dilemma that arises when families attempt to “protect” the patient by withholding the diagnosis and other information, and treatment decisions are typically made by a family member.

**Table 4:** Examples of ethical concerns discussed

Ethical concern	Example
Conflict of interest	Should consultants charge medical students, trainees, nurses or fellow consultants for clinic visits or procedures?
Confidentiality	Is it ethical to disclose patient information to someone in the hospital who is not a member of the healthcare team?
Consent taking	Is it sufficient to inform a patient about the physical examination by a resident/student or do we also need to give them the option to refuse by phrasing it as a question?
Resource allocation	Provincial government develops a plan without the involvement of the health teams at the district level
Harassment	Junior doctors being bullied by consultants, name calling in front of the patients
Do no harm	An attending physician suggesting surgery when it is not necessary
Policies not being followed	Psychiatry patients not allowed to change their doctor or take LAMA (leave against medical advice) despite the hospital’s policy on patient’s rights and responsibilities
Professionalism	Poor role modelling – attending physicians wear gowns, scrubs, shoe covers, etc. in and out of the OR, but students and trainees are questioned about the same

#### Nature of participation in “Let’s Talk” forum

While learners posted actively during the first month of the intervention, the number gradually declined. Learners mentioned that they could not utilise EthAKUL regularly due to clinical responsibilities and exams. Conversely, facilitators speculated that the drop in participation might be because m-JiTL required deep reflection in/on practice, and learners were unfamiliar with the new learning strategy: “The app is developing a new culture. It may be possible that people are still reluctant to learn [in] the new virtual discussion forum” (F2, Female). Another facilitator mentioned that because the posts were visible to everybody, learners who were lurking (that is., reading but not posting), were able to understand what some of the ethical concerns were and how they may be responded (Female, F2). It was proposed that the learners be encouraged to post their comments, and that facilitators foster critical thinking by asking probing questions and summarising the conversation.

#### *Facilitation of learning *via* EthAKUL*

Facilitation of discussion was mentioned as a challenge by both learners and facilitators. The learners were disappointed that the facilitators did not give answers on how to respond to the ethical problems and that the discussion threads remained open-ended with no conclusions. The facilitators agreed that the “Let’s Talk” forum had devolved into a question-and-answer session, with learners expecting facilitators to respond to their queries rather than engaging in a dialogue and questioning and expanding on others’ views:

“There is no closure. You don’t feel like you have done your job. Have they understood or not? One is not sure if [the ethical dilemma] has been resolved, or has the thinking process changed, or was it what the person expected? They do not have to agree with [the response]. But did they process it?” (F4, Female).

The facilitators were concerned about a small number of postings and one facilitator (F3, Female) commented that due to a lack of body language and other visual clues it was impossible to tell if the students had grasped the principles. They commented that learners needed more help understanding the fundamental bioethics principles, identifying ethical dilemmas in clinical settings, and describing dilemmas in a written format, and these areas could be addressed in the taught sessions.

### Future use of EthAKUL

The facilitators agreed that “[the] app by itself is not an effective teaching tool until it is mixed in a face-to-face classroom context” (F6, Male). It was proposed that EthAKUL be utilised as part of a mixed strategy to reinforce key principles in post-graduate medical education, where before and after the lesson, students can debate ethical concerns. EthAKUL can also be used during clinical rotations in undergraduate clinical years (Years 3–5), and nursing. EthAKUL was suggested as a tool for promoting inter-professional conversations among physicians, nurses, and other members of the healthcare community. EthAKUL, according to one facilitator, might be a “beginning point or foundation for institutions with no bioethics competence” (F7, Female). In other words, facilitators regarded EthAKUL as a mechanism to facilitate bioethics teaching in medical schools that lacked relevant expertise.

## Discussion

The study was aimed at determining EthAKUL's effectiveness and feasibility for curriculum-wide adoption at a university in Pakistan. The quantitative results show an improvement in learners’ bioethics knowledge, with a notable improvement in the medical student cohort. Also, learners’ attitude towards m-Learning remained largely positive after the intervention. Nevertheless, the qualitative and quantitative data show discrepant findings regarding improvement in bioethics knowledge and learner experiences. The “Let’s Talk” forum was the most popular feature, and access to policies and anonymous posting were deemed advantageous. There was also a disparity in perceptions of the purpose of the “Let’s Talk” forum between facilitators and learners, and several posts were not related to the modules in EthAKUL. Though meant to be used for m-JiTL, over a third of students (37.9%) used EthAKUL once every two weeks or once a month, and the vast majority utilised it on weekends, with only a small percentage using it during work hours. Finally, limited participation as evidenced by a small number of posts, lack of closure in discussions, and the absence of certain app features posed challenges that need to be addressed.

As younger health professionals are considered early technology users, the low response rate in technology-related research is unexpected [16], but it is comparable to other similar studies [18, 25, 39]. Low participation in technology-based intervention studies has been attributed to several reasons, including a reluctance to participate in research projects during early career transitions and low priority given by medical and nursing students because it is not formally counted in teaching and learning assessments [16, 24]. In our study, the low response rate (41%) could be because the post-intervention tools were emailed to the learners, and email surveys generally received low responses [40]. Declining participation could be because EthAKUL was an optional activity, not a part of the assessment. The facilitators also acknowledged lurking [41] by some participants. However, due to the lack of usage logs, we were unable to determine whether participants were lurking, or they had stopped using EthAKUL due to conflicting priorities in an environment where assessment drives learning. The reasons for not posting on the discussion forum could be explored in future studies.

The facilitators recognised that the m-JiTL pedagogy was different from what the learners were used to in didactic sessions. The m-JiTL pedagogy depended on learners initiating asynchronous online discussions on the “Let’s Talk” forum during their clinical rotations. Learners had to first identify an ethical dilemma, post it on the forum and engage in a conversation with peers and facilitators for learning to take place. In short, they were required to reflect in-action and on-action [42] and interact in real-time with peers and faculty. The facilitators were expected to nudge and probe, be more engaged, foster reflections, and synthesise information instead of directing the conversation [43]. Asynchronous discussions also necessitated facilitators relinquishing their role as the sage on the stage and assuming the role of a guide on the side, which differed from didactic teacher-led pedagogy commonly used in bioethics teaching. In healthcare systems in Pakistan, where consultants (or teachers) maintain positions of authority, this teaching adjustment required a shift in power relations between teacher and learners [44], which was challenging to achieve in the project. A community of inquiry model can encourage meaningful exchanges and reflections in asynchronous online discussions [45]. Previous studies have shown that to create a community of inquiry, asynchronous discussions in graduate online bioethics courses should emphasise cognitive, social, and instructional presence [46]. Learners should receive timely and detailed feedback on their discussion performance, and there should be chances for learners to engage in social interactions [46].

The role of language proficiency in learners’ participation in discussion-based teaching cannot be ignored. In the current study, written English proficiency may have hampered participation in text-based discussions. Although research has shown that text-based discussions can boost English as Second Language (ESL) users’ engagement by offering more time to write [47], the pedagogy in the course required just-in-time discussions, which may not have given the learners enough time to think and write. Moreover, research indicates that some ESL users prefer speaking to writing due to their language proficiency [48, 49]. Previous research has also identified distinct advantages of audio-discussion [50]. As a result, in future iterations of EthAKUL, the option of audio-discussions could be included to support learners who had difficulty expressing themselves in writing.

The learners’ use of EthAKUL during non-clinical time is consistent with previous research [51] and serves as a reminder that the teaching approaches must be compatible with the discipline’s pedagogy. Bioethics teaching and learning requires learners’ reflection on the issues [52] and adapting teaching to support a deeper understanding of ethical dilemmas. Further, the disconnect between the concepts presented in the standard bioethics curriculum and the learners’ real experiences observed through the posts reaffirms that students’ “… ethical domain may be broader than ethical dilemmas and conflicting choices, and includes definitions focused on responsibilities, emotions, notions of justice and quality of care” [53]. Within an appropriate pedagogical framework, the app can be used to address learners’ broader ethical domain and facilitators need to be aware of clinical scenarios that trainees view as ethical challenges, and the curriculum should be updated regularly to address learners’ needs.

In the current study undertaken before the COVID-19 pandemic, the use of m-JiTL involved an interprofessional approach involving medical and nursing students and residents. The pandemic has underscored the need for purposefully designed technology-enhanced learning systems [54]. The learners’ positive attitude toward mobile learning may work as a motivation to make learning materials and policies accessible and create discussion forums to incorporate bioethics information into practice [31]. However, some bioethics teaching issues may be due to generational differences, facilitators' approaches to teaching and learning [55, 56], or learners' reluctance to ask questions or continue a discussion due to linguistic competence [48]. When the pedagogical approaches challenge conventional roles and hierarchies, student and faculty preparation is even more crucial. The findings highlight the significance of faculty and student preparation, particularly regarding implementing m-JiTL across the curriculum. The study also suggests that m-JiTL should be strategically implemented within a blended learning framework, with students serving as co-creators of the curriculum. Further research on m-JiTL that involves learners in co-creating learning experiences in various settings is recommended.

The study has certain limitations that can guide future research. Firstly, the number of participants was small because of the app limitation and our need to test the new teaching approach with a small number of learners before the curriculum-wide adoption and make it available to others. Due to the limited server capacity, we could only have a maximum of 100 users (including learners, facilitators, and researchers). Second, the participants belonged to a private university in Karachi that actively promotes bioethics teaching and technological integration into medical and nursing education. Third, despite the positive perceptions suggested by the self-reported data, the intervention received only a modest response rate and level of participation. Due to the absence of usage logs, the researchers could not determine how many participants had continued using the mobile app, though they did not respond to the post-questionnaire or post-test. Additionally, this study assessed learners’ performance on bioethics knowledge tests before and after the intervention, which revealed an improvement. However, because the project was an optional activity, we cannot conclude for certain that the improvement was only a result of the intervention. It is possible that the learners were intrinsically motivated to learn more about bioethics and had access to resources other than those provided in the intervention. Moreover, we did not investigate changes in bioethics practise, which could be studied in future interventions. Finally, the findings of this study cannot be extended to other medical and nursing education institutions in Pakistan or other LMICs, and there is a need for additional empirical research in various institutional contexts.

## Conclusion

In conclusion, as the first study to test a custom-designed mobile app and m-JiTL approach for teaching bioethics in an LMIC such as Pakistan, the findings suggest that new approaches to bioethics education have promise. This study generated several practical suggestions related to the mobile app features and pedagogy of m-JiTL for teaching bioethics. However, the study also revealed that mobile apps must be aligned with the required curricular outcomes and only be implemented if faculty and students are prepared for the new teaching and learning strategy. The findings also suggest that a blended approach to teaching bioethics may be more effective, where m-JiTL complements didactic teaching. The bioethics educators who intend to implement m-JiTL should carefully consider the learning outcomes, pedagogy, and assessment through curriculum alignment and robust faculty and student readiness. Finally, we propose more nuanced interventions in a variety of contexts to investigate EthAKUL's bioethics curriculum-wide integration.

## Data Availability

Data collection tools available on request. The datasets generated and analysed during the current study are not publicly available as they contain student demographics and teaching data, but a de-identified version is available from the corresponding author on reasonable request.
